# Long-Term Outcome and Predictors of Transversus Abdominis Plane Block for Chronic Post-Hernioplasty Pain

**DOI:** 10.3390/jcm13144039

**Published:** 2024-07-10

**Authors:** Ulderico Freo, Maurizio Furnari

**Affiliations:** Institute of Anesthesia and Intensive Care, Department of Medicine—DIMED, Padua University Hospital, Via Giustiniani 2, 35121 Padua, Italy

**Keywords:** hernia repair, chronic post-herniorrhaphy pain, transversus abdominis plane block

## Abstract

**Background/Objectives**: Different analgesic techniques have been used in the clinical management of chronic post-hernioplasty pain (CPHP), with variable results. This study aimed to investigate clinical factors associated with long-term outcome of the transversus abdominal plane (TAP) block for CPHP. **Methods**: We retrospectively analyzed 26 patients with CPHP who were treated with single or multiple TAP blocks with local anesthetic and steroid. Patients were evaluated for pain and neuropathic pain intensity by a Numerical Rating Scale (NRS) and the painDETECT questionnaire (PDQ), for anxiety and depression by the Hospital Anxiety and Depression Scale, and for quality of life by the 12-item Short Form Health Survey (SF12). **Results**: At 6 months post-treatment, 20 patients (77%) presented substantial (>50%) or moderate (30–50%) CPHP relief and were considered responders. In responders, the 24-h average and maximum NRS pain significantly declined (*p* < 0.01) from 7.3 ± 1.3 to 2.6 ± 2.1 and from 8.8 ± 1.5 to 5.1 ± 2.0, and the neuropathic PDQ score from 9.1 ± 3.2 to 6.1 ± 1.3; the physical SF12 score improved from 36.5 ± 5.8 to 44.3 ± 7.5 (*p* < 0.01). Six patients failed to achieve a significant CPHP improvement and were considered non-responders. Non-responders presented a significantly (*p* < 0.05) longer CPHP, higher body mass index and neuropathic symptoms, and more frequent anxiety, depression, diabetes, and fibromyalgia. **Conclusions**: The TAP block with local anesthetic and steroid should be considered as a therapeutic option for CPHP. However, medical and psychiatric comorbidities negatively impact the TAP block effectiveness for CPHP.

## 1. Introduction

Abdominal wall hernia repair is one of the most common surgical procedures, being performed in over 20 million people annually, worldwide [1,2,3]. Acute pain after surgery is usually expected to subside within a few days or weeks; chronic, i.e., longer than 3 months, post-hernioplasty pain (CPHP) occurs in 10–12% of patients, causing disability and the need for medical treatment in 1–3% of patients [1].

The transversus abdominis plane (TAP) block is a regional anesthesia technique that requires the injection of local anesthetics into the fascial plane, between the internal oblique and the transversus abdominis muscles [2,3,4,5,6,7,8]. Since the original description in 2001 and the later description of the ultrasound (US) technique, TAP blocks have become very popular in providing postoperative analgesia in a variety of laparoscopic and open abdominal surgeries including the repair of inguinal hernia and abdominal wall defects [2,3,4,5,6,7,8]. Patients who underwent hernia repair under a TAP block reported less postoperative pain and opiate requirement and improved mobility [2,3,4,5,6,7,8]. The addition of buprenorphine, dexmedetomedine, magnesium, or steroids to the local anesthetic further enhanced the quality of the TAP block and postsurgical recovery [9,10,11,12]. 

However, the use of the TAP block for inguinal hernia repair had no or minimal preventive effect on subsequent CPHP [2,6,7]. Also, the use of the TAP block to treat CPHP yielded variable outcomes [13,14,15,16]. We therefore aimed to investigate long-term efficacy and clinical predictors of TAP block for CPHP.

## 2. Patients and Methods

Patients with CPHP were referred to the Pain Therapy of the Anesthesiology and Intensive Care Institute of Padua University Hospital (Padua, Italy). Clinical data were recorded as described previously and included demographics, medical and surgical comorbidities, pain disorders (i.e., fibromyalgia, irritable bowel disease, migraine, arthritis, and inflammatory osteoarthritis), and medications [17]. 

Pain intensity was quantitated as 24 h average pain at rest and maximum pain at movement by a 0–10 pain verbal Numerical Rating Scale (0 = no pain, 10 = worst imaginable pain, NRS). Neuropathic pain symptoms were assessed with the painDETECT Questionnaire (PDQ), which is a 9-item self-administered questionnaire with a total score ranging from −1 to 38 and cut-offs to distinguish between neuropathic and other non-neuropathic pains (PDQ scores: ≤12 = nociceptive pain; scores 13–18 = unclear, mixed pain; scores of ≥19 = high likelihood of neuropathic pain). Anxiety and depression symptoms were determined by the Hospital Anxiety and Depression Scale (HADS), which is a 14-item questionnaire with cut-offs for severity (scores 0–7, normal; scores 8–10, 11–14 and 15–21 indicative of, respectively, mild, moderate and severe anxiety or depression). Mental and physical quality of life was evaluated by the 12-item Short-Form survey (SF12) [17] with cut-offs for disability level (scores > 50, no disability; scores 40–50, 30–40 and <40, indicative of mild, moderate and severe disability). Patients were considered responders if, at 6 months after the TAP block, they had substantial pain relief (>50%) or moderate relief (30 to 50%) and non-responders if they did not experience significant pain relief (<30%). 

Patients were treated with a TAP block after an assessment by a general surgeon and radiological investigations had ruled out hernia recurrence and complications, and after multimodal pharmacological therapies (i.e., non-steroid anti-inflammatory drugs, opioids, gabapentinoids, and noradrenergic antidepressants) had failed or were not tolerated. 

For the TAP block procedure, patients were placed in the supine position and the abdominal skin was prepared with a 5% povidone iodine solution and covered with sterile drapes; the ultrasound probe was placed obliquely near the midline over the anterior abdominal wall and along the subcostal margin and was moved laterally to identify the neurofascial plane between the rectus and transversus abdominis muscle. Injections were performed with a 22-gauge 3.5-inch needle that was advanced in the plane of the US beam to approach the TAP plane. The injection site was anesthetized using lidocaine 1% and then, after negative aspiration, a solution of lidocaine 0.5% and ropivacaine 0.35% and 40 mg of triamcinolone was injected into the TAP under direct US visualization, ensuring the spread of the medication into the appropriate plane. The needle was then flushed with lidocaine 1% and withdrawn. The TAP block was performed 1 to 4 times, approximately 15 days apart.

Data are presented as means ± standard deviations, and numbers (and percentages) of patients. Normality was assessed with the Shapiro–Wilk test. Normally distributed data were analyzed by a *t*-test and a one-way analysis of variance (ANOVA); non-normally distributed data were analyzed using Mann–Whitney U, Kruskal–Wallis, and Friedman tests. Categorical variables were assessed with a Chi-square test. Bonferroni correction was applied to adjust for multiple comparisons. Differences were considered statistically significant for values of *p* < 0.05. All statistical tests were performed in GraphPad 10.2.2.

## 3. Results

Twenty-six CPHP patients (20 women and 6 men) underwent one to four TAP blocks for a total of 59 procedures. Demographic and clinical features are presented in Table 1. 

**Figure 1:**
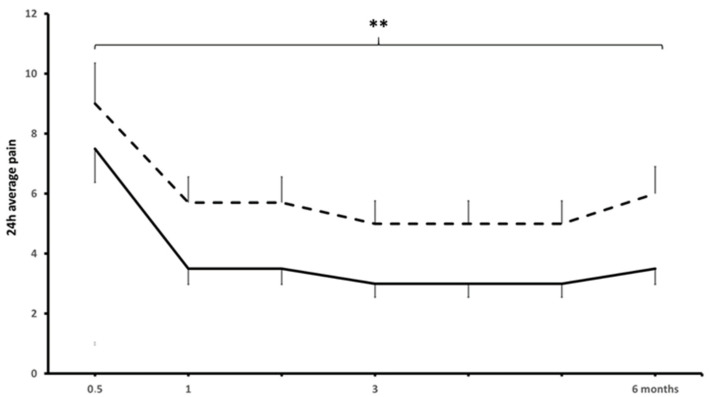
Twenty-four-hour average (continuous line) and worst NRS pain (broken line) in CPHP patients from pre-treatment to 6 months post-treatment; different from baseline, **, *p* < 0.01.

The TAP block provided clinically meaningful, long-lasting CPHP relief in 77% of patients (*p* < 0.01) (Figure 1). From pretreatment to post-treatment, the 24 h average and worst pain declined from 7.2 ± 1.5 to 3.5 ± 2.0 (a 48.1 ± 32.7% decrease) and from 9.0 ± 1.1 to 6.1 ± 2.3 (a 30.6 ± 23.4% decrease) (Figure 1). Fourteen patients experienced substantial pain relief (24 h average pain from 7.1 ± 1.5 to 2.5 ± 2.0 for a 71.6 ± 8.2% reduction) and six patients experienced moderate pain relief (from 7.6 ± 2.1 to 4.8 ± 2.1 for a 35.8 ± 2.5% reduction) and were considered responders; six patients did not achieve clinically meaningful pain relief (5.6 ± 3.5%) and were classified as non-responders. At pretreatment, the NPQ score was consistent with neuropathic, mixed, and nociceptive pain in 1, 12, and 13 patients. From pre-treatment to post-treatment, NPQ declined significantly (10.8 ± 5.3 to 7.7 ± 4.8 for an overall reduction of 23.1 ± 31.1, *p* < 0.05) and to a larger degree in responders compared to non-responders (26.8 ± 25.5% vs. 13.6 ± 6.3%, *p* < 0.05) (Table 2). 

Pretreatment anxiety and depression HADS scores were 6.7 ± 3.4 and 6.5 ± 3.5, respectively, and were consistent with mild or moderate anxiety or depression in five and six patients; HADS scores were not significantly modified. Pretreatment SF12 physical and mental scores (37.1 ± 6.3 and 47.0 ± 6.0) were consistent with mild, moderate, and severe physical disability in five, nine, and three patients and mild-to-moderate mental disability in thirteen patients. At the post-treatment follow-up, the SF12 physical score improved (7.0 ± 8.2 points, *p* < 0.01) more in responders than non-responders (8.0 ± 6.8 vs. 3.4 ± 4.5 points, *p* < 0.01); seven responders recovered to normal SF physical scores (>50). Mental SF12 was not significantly altered.

CPHP patients responders and non-responders to TAP block did not differ in age, gender distribution (Table 1), or mean numbers of previous abdominal surgeries (1.3 ± 1.7 and 1.8 ± 1.8), non-abdominal surgeries (0.7 ± 1.5 and 0.8 ± 1.2), and in numbers of TAP blocks (2.3 ± 0.9 and 2.3 ± 1.0) they had undergone. Non-responders, however, presented significantly more medical comorbidities, more intense neuropathic pain, more frequent anxiety, depression, diabetes, fibromyalgia, and obesity, and longer-lasting CPHP (Table 1 and Table 2).

## 4. Discussion

CPHP affects daily activities in 5% to 10% of patients after herniorrhaphy; CPHP is ascribed to perioperative lesions of the anterior abdominal wall due to surgical manipulation, scarring, or inflammation. Treatment options include watchful waiting, systemic analgesics, injections of local anesthetics for regional anesthesia or at tender points, and radiofrequency of the abdominal wall nerves (i.e., ilioinguinal, iliohypogastric, and/or genitofemoral nerves); if treatments fail, triple neurectomy of the inguinal nerves and mesh removal are considered [1,5].

Insofar, regional anesthesia techniques provided variable results for treating CPHP. In a controlled-versus-placebo crossover trial, a US block with lidocaine of the ilioinguinal and iliohypogastric nerves failed to give significant CPHP relief [13]. A TAP block with bupivacaine and triamcinolone for chronic abdominal wall pain in patients with a history of previous surgery (75 patients, 26 hernia repairs) or without previous surgery (17 patients) gave an average of 50% pain relief for 108 days in 82% of patients [14]. Baciarello and coworkers reported the effects of a TAP block with bupivacaine and triamcinolone in five patients with chronic abdominal pain; two patients reported low-intensity pain at 6- and 12-month follow-ups [15]. Hence, the 77% effectiveness of the TAP block for CPHP reported in the present study is similar to previous studies; the 6-month benefit time extends previous observations and reports [13,14,15,18]. The study also confirms that the TAP block can improve or normalize the SF12 physical quality of life in significant numbers of patients who were able to resume their personal, social, and working activities, thus reducing the economic burden related to CPHP [18].

In addition, the TAP block improved neuropathic symptoms. Although it may be difficult to apply the diagnostic grading algorithm for neuropathic pain in CPNP, intense neuropathic symptoms and sensory abnormalities are common in CPHP and can be estimated by the PDQ [17,19]. The strong association between sensory abnormalities and pain suggests the significant role of neuropathic mechanisms in chronification processes of CPHP [20]. As neuropathic pain is associated with higher degrees of anxiety, depression, and sleep disorders, it is worth noting that the TAP block was effective in attenuating this particularly bothersome symptom of CPHP [20]. 

Risk factors for CPHP include young age, female gender, previous pregnancy, oral contraceptives, high preoperative and postoperative pain, recurrent hernia, mesh weight, and open repair [1,9]; other factors that have been implicated in unsatisfactory analgesic responses to the TAP block were nerve anatomical variations or previous surgeries [13,14]. In our study, however, anthropometric variables and the number of surgeries did not appear to impact TAP block efficacy. In contrast, non-responders presented higher symptoms and numbers of medical and psychiatric conditions (i.e., diabetes, obesity, fibromyalgia, and depression), more intense NPQ neuropathic pain, and longer CPHP.

Psychological factors such as anxiety and depression reduce the pain threshold and increase attention and hypervigilance to pain and the risk of chronic postoperative pain [21,22]. Diabetes and obesity may cause chronic, low-grade, diffuse inflammation and increase the risk of diffuse musculoskeletal and neuropathic pain [21,22,23,24,25]. Further, chronic pain and chronic neuropathic pain result from different mechanisms. However, regardless of its origin, chronic pain including primary pain conditions (i.e., fibromyalgia) favors processes of peripheral and central sensitization; the latter, in turn, may impair the descending inhibitory systems and contribute to a slower resolution and chronification of postoperative pain [25]. These findings confirm previous studies and suggest that patient profiling and prompt treatment of postoperative pain are essential to prevent pain chronification and CPHP [22,24].

Our study has several limitations. First, it is retrospective in nature and was conducted on a small number of patients from a single center; second, patients underwent variable numbers of TAP blocks; third, the lack of a presurgical evaluation and healthy controls prevents us from assessing the impact of pre-existing psychological conditions and the placebo effect on the response to the TAP block.

In conclusion, however, the TAP block induced a meaningful and long-lasting improvement in pain and quality of life in 77% of CPHP patients; medical and psychiatric comorbidities were associated with the failure of the TAP block.

## Figures and Tables

**Table 1:** Demographic and clinical features of CPHP patients treated with TAP block.

	All	Responders	Non Responders
Patient number	26	20	6
Age, y	56.5 ± 12.2	58.1 ± 12.5	53.4 ± 10.0
Height, cm	171.6 ± 8.4	170.6 ± 9.3	173.4 ± 7.6
Weight, kg	77.2 ±14.5	72.9 ± 13.5	88.3 ± 15.7
M/F	20/6	15/5	5/1
BMI	26.2 ± 3.3	24.9 ± 2.8	29.2 ± 3.8 *
Pain duration, months	26.4 ± 21.7	16.8 ± 16.1	49.4 ± 37.5 **
Comorbidities	1.8 ± 0.5	1.3 ± 0.3	3.2 ± 0.7
Arthritis	6	3 (15)	3 (50)
Cancer	6	6 (30)	0
Depression	8	3 (15)	5 (83) *
Diabetes	3	0	3 (50) **
Fibromyalgia	4	0	4 (67) **
Osteoporosis	3	1 (5)	2 (33)
Hypertension	8	4 (20)	4 (67)
Obesity	2	0	3 (30) *
Osteoporosis	1	1 (5)	2 (33)
Thyroid disorders	5	2 (10)	3 (50)

Data are means ± SD or numbers of patients (%); *^,^ **, different from responders, *p* < 0.05 and *p* < 0.01. BMI, body mass index; M/F, male/female; y, years.

**Table 2:** Neuropathic, anxiety, and depressive symptoms and quality of life in responders and non-responders to TAP block for CPHP.

	Responders		Non Responders	
	Baseline	Follow Up	Baseline	Follow Up
PDQ	9.6 ± 6.2	5.8 ± 2.7 ^#^	14.5 ± 9.1 *	13.3 ± 5.8
HADS, anxiety	5.6 ± 2.6	4.5 ± 3.2	9.9 ± 3.2 **	9.6 ± 3.4
HADS, depression	6.0 ± 1.6	5.6 ± 3.2	9.8 ± 3.3 **	8.3 ± 2.6
SF-12, physical	37.1 ± 6.3	47.3 ± 6.3 ^##^	33.4 ± 6.0	37.6 ± 5.6
SF-12, mental	46.3 ± 6.3	48.8 ± 6.3	41.4 ± 4.9	41.6 ± 4.1

HADS, Hospital Anxiety and Depression Scale; PDQ, painDETECT questionnaire; SF-12, 12-Item Short-Form Health Survey. Data are means ± SD; *^,^**, different from responders, *p* < 0.05 and *p* < 0.01; ^#, ##^, different from baseline, *p* < 0.05 and *p* < 0.01.

## Data Availability

Data are contained within this article.
